# Supplementary material for: The interactome of intact mitochondria by cross-linking mass spectrometry provides evidence for coexisting respiratory supercomplexes
Source: Mol Cell Proteomics. 2017 Dec 8;17(2):216–32. doi: 10.1074/mcp.RA117.000470 (PMC5795388; doi:10.1074/mcp.RA117.000470)
Supplement: Supplemental Data [file supp_RA117.000470_133922_0_supp_23978_fzffwf.zip › spectra_annotation/mito_DR_spectra_annotation/318-1-15-1-15-1.pdf]

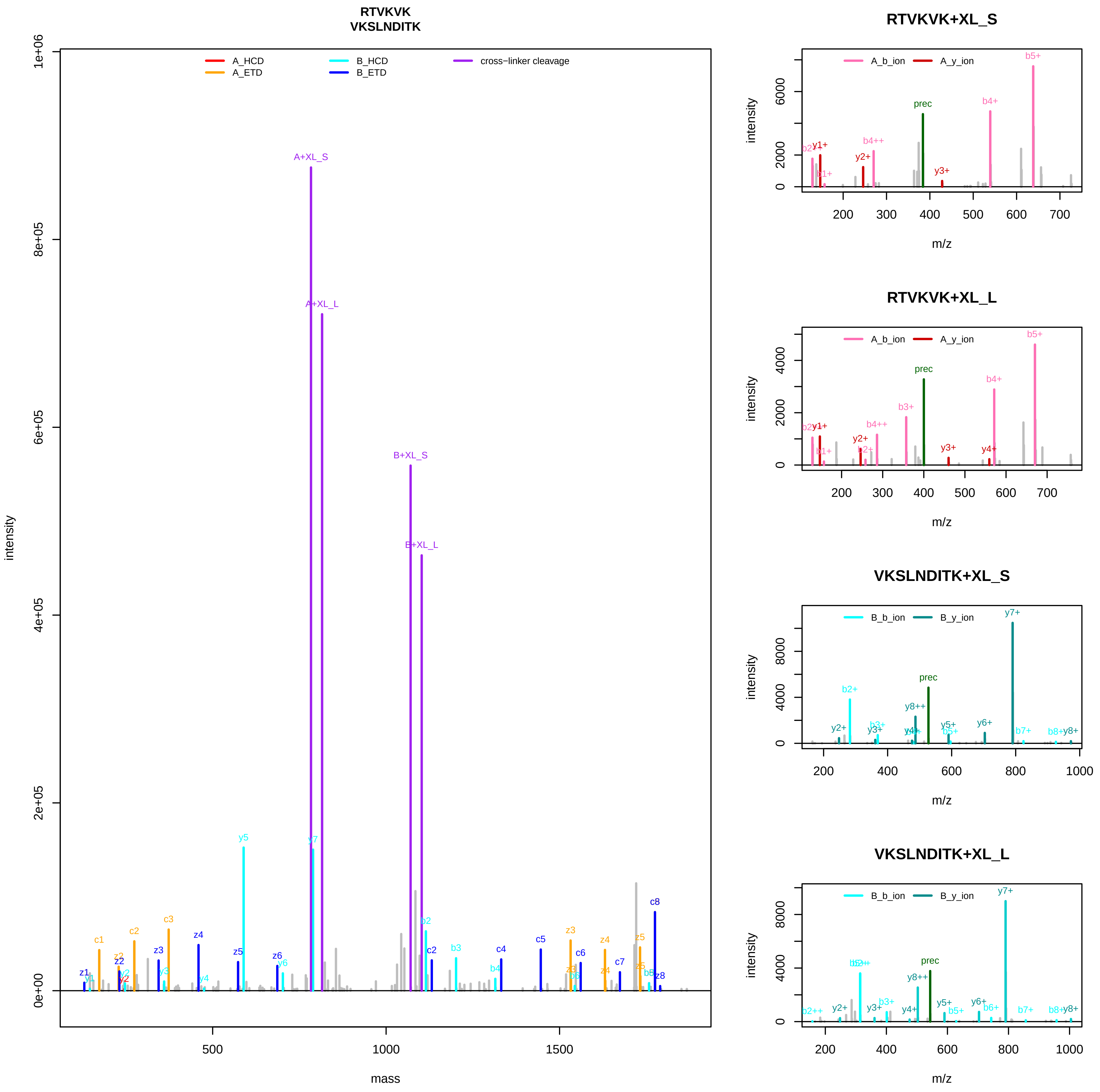

RTVKVK
VKSLNDITK
A_HCD
A_ETD
B_HCD
B_ETD
cross−linker cleavage
A+XL_S
A+XL_L
B+XL_S
B+XL_L
y5
y7
b2
c8
z1
c1
c2
c3
z2
y2
z3
y3
z4
y4
z5
z6
y6
c2
b3
c4
b4
c5
z3
c6
z4
c7
z5
b8
z8
intensity
mass
0e+00
2e+05
4e+05
6e+05
8e+05
1e+06
500
1000
1500
RTVKVK+XL_S
A_b_ion
A_y_ion
b5+
b4+
prec
b4++
y1+
y2+
y3+
b1+
intensity
m/z
RTVKVK+XL_L
A_b_ion
A_y_ion
b5+
b4+
prec
b3+
b4++
y2+
y3+
y4+
b1+
intensity
m/z
VKSLNDITK+XL_S
B_b_ion
B_y_ion
y7+
prec
b2+
y8++
y2+
y3+
b3+
y4+
y5+
b5+
y6+
b7+
b8+
y8+
intensity
m/z
VKSLNDITK+XL_L
B_b_ion
B_y_ion
y7+
prec
y8++
b2++
y2+
y3+
b3+
y4+
y5+
b5+
y6+
b6+
b7+
b8+
y8+
intensity
m/z
